# Chloroform Administration

**Published:** 1889-04

**Authors:** 


ARTICLE VII.

### CHLOROFORM ADMINISTRATION.

At the distribution of the prizes to the students of the Hyderabad Medical School, by their Royal Highness the Duke and Duchess of Connaught, on January 25th, Surgeon-Major Lawrie, M. D., Principal of the Medical School, in a short address, referred to the commission appointed last year by the Nizam's Government to make experiments with reference to the effects of chloroform. Dr. Lawrie said the experiments which had been carried out by the commission, consisting of Dr. Hehir, Mr. Kelly and Mr. Chamarette, were, in his opinion, the most important that had ever been made, and had conclusively decided a question which had been in dispute ever since chloroform was first introduced. There was no doubt that the anæsthesia produced by chloroform was best measured by its effect on the breathing, and that when the administration was pushed beyond a safe point, the breathing became embarrassed and then stopped. The question in dispute was whether chloroform ever affected the heart directly or not; and this was important in its bearing on the way in which the administration of the anæsthetic should be conducted. The following was the work performed by the commission, as described by Dr. Lawrie. They killed with chloroform 128 full-grown pariah dogs, averaging over twenty pounds weight each. This does not represent a tithe of the experiments they actually performed, which really amounted to several hundreds, as they varied the dose and the method of administering the chloroform in every possible way, and tested the value of artificial respiration in nearly every case by reviving the dogs over and over again after the breathing had stopped, and before the heart ceased beating. What they found was, that no matter in what way it was given, in no case did the heart become dangerously affected by chloroform until after the breathing had stopped. "This," adds Dr. Lawrie, "tallies exactly with my own experience.

I have given chloroform as often, or oftener, than any man living, and have never had a fatal case; and I can state positively that in the 40,000 or 50,000 administrations I have superintended I have never seen the heart injuriously or dangerously affected by it. I take no credit to myself in this matter. I have simply carried out in India the principles Simpson and Syme practised and taught in Edinburgh." In the hospitals attached to their school, chloroform was invariably given with absolute, or, he might almost say, with guaranteed safety, by students, and they were never allowed to examine the heart beforehand, or feel the pulse during its administration. In other places, and in London itself, deaths from chloroform constantly occurred, but provided the administration could swear he examined the heart and felt the pulse, they were always supposed to be accidental. He, Dr. Lawrie, had no doubt deaths would go on occurring until the London schools, which of course influenced the whole world, either entirely changed their principles and ignored the heart in chloroform administration, or else confined themselves exclusively to the use of an anæsthetic like ether, which, with all its disadvantages, they knew how to manage.—*British Medical Journal.*

